# Metabolic changes during pregnancy in glucose‐intolerant NZO mice: A polygenic model with prediabetic metabolism

**DOI:** 10.14814/phy2.14417

**Published:** 2020-05-06

**Authors:** Katharina Grupe, Melissa Asuaje Pfeifer, Franziska Dannehl, Moritz Liebmann, Ingo Rustenbeck, Annette Schürmann, Stephan Scherneck

**Affiliations:** ^1^ Institute of Pharmacology, Toxicology and Clinical Pharmacy Technische Universität Braunschweig Braunschweig Germany; ^2^ Department of Experimental Diabetology German Institute of Human Nutrition Potsdam‐Rehbrücke Nuthetal Germany; ^3^ German Institute for Diabetes Research (DZD) Neuherberg Germany

**Keywords:** impaired glucose tolerance, polygenic mouse model, prediabetes, pregnancy

## Abstract

Gestational diabetes mellitus (GDM) is a complex metabolic disease involving genetic and environmental factors. Recent studies have underlined its heterogeneity, so it is reasonable to divide patients into subpopulations depending on whether an insulin secretion or sensitivity defect is predominant. Since testing for GDM is usually performed in the second trimester, misinterpretation of prediabetes as gestational diabetes may occur. As with type 2 diabetes (T2DM), rodent models are needed for both GDM and prediabetes, but few do exist. Here, we compared the metabolic changes in pregnant normal NMRI mice with those in New Zealand obese (NZO) mice. Male animals of this strain are an established model of T2DM, whereas female mice of this strain are protected from hyperglycemia and β‐cell death. We demonstrate that female NZO mice exhibited impaired glucose tolerance, preconceptional hyperinsulinemia, and hyperglucagonemia without any signs of manifest diabetes. The NZO model showed, compared with the NMRI control strain, a reduced proliferative response of the Langerhans islets during pregnancy (3.7 ± 0.4 vs. 7.2 ± 0.8% Ki‐67‐positive nuclei, *p* = .004). However, oral glucose tolerance tests revealed improved stimulation of insulin secretion in both strains. But this adaption was not sufficient to prevent impaired glucose tolerance in NZO mice compared with the NMRI control (*p* = .0002). Interestingly, glucose‐stimulated insulin secretion was blunted in isolated primary NZO islets in perifusion experiments. In summary, the NZO mouse reflects important characteristics of human GDM and prediabetes in pregnancy and serves as a model for subpopulations with early alterations in glucose metabolism and primary insulin secretion defect.

## Graphical abstract

The aim of this work was to evaluate metabolic changes during pregnancy within the polygenic NZO mouse model and if it is a suitable one for the human disease. NZO mice showed early alterations in glucose homeostasis and insulin secretion, but no overt diabetes. Interestingly, the preconceptionally impaired glucose tolerance did not deteriorate during gestation despite a proliferation defect of Langerhans islets.

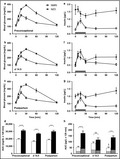

## 1 INTRODUCTION

Gestational diabetes mellitus (GDM) is defined as any degree of glucose intolerance with first onset or recognition during pregnancy, diagnosed between the second or third trimester and clearly differentiates from a preconceptionally existing manifest diabetes (American Diabetes Association, 2013, 2019). However, the clinical definition disregards the fact that screening also classifies women as GDM patients who already had an undiagnosed preconceptional impaired glucose tolerance but no manifest diabetes. The mechanisms involved are heterogeneous and range from impaired β‐cell function to insulin resistance with hyperinsulinemia. GDM is a complex metabolic disease in which both environmental or lifestyle factors and a genetic predisposition are involved. There is evidence from observational studies that modifiable factors like pregravid adiposity, physical activity, and dietary patterns are involved in pathogenesis (Zhang & Ning, 2011).

During normal pregnancy, hormonal and metabolic alterations are observed to meet maternal and fetal demands, which furthermore undergo changes as pregnancy progresses. Normally, insulin sensitivity is unchanged or increased in first trimester and resistance increases in the second or third trimester to supply glucose for the developing fetus (Di Cianni, Miccoli, Volpe, Lencioni, & Del Prato, 2003; Stanley, Fraser, & Bruce, 1998). This is compensated by adaptive mechanisms of the Langerhans islets, which increase both basal and glucose‐stimulated insulin secretion during pregnancy (Catalano, 1994; Sorenson & Brelje, 1997). The conventional view on GDM in recent decades has been that the disease is caused by a lack of β‐cell adaptation and a consequential insufficient insulin secretion. Besides secretory defects, reduced insulin receptor binding and postreceptor signaling defects were considered to be related to the development of GDM (Catalano, 2014; Devlieger, Casteels, & Van Assche, 2008; Kühl, Hornnes, & Andersen, 1985). Due to the heterogeneity of diabetes, subdivision into the different types (T1DM, T2DM, GDM, etc.) is often not sufficient. As a consequence, a more detailed characterization of these patients seems reasonable and stratification of T2DM patients might further contribute to improved and targeted treatment (Ahlqvist et al., 2018; Tuomi et al., 2014). Subtypes of GDM were also characterized and it was shown that GDM is a heterogeneous disease as well, depending on whether an insulin secretion or sensitivity defect is predominant (Powe et al., 2016). Moreover, it could be shown that insulin response increases independently of changes in insulin sensitivity in early pregnancy, which might be associated with pregnancy‐related hormonal changes (Powe, Huston Presley, Locascio, & Catalano, 2019).

To study the pathomechanisms of GDM, numerous animal models are described. These range from surgical and chemically induced strategies to nutritional and genetic manipulations (Pasek & Gannon, 2013). Models showing the phenotype of gestational diabetes are often based on knockout strategies, for example, monogenic models such as the db/+, PrlR^±^, or FoxM1^Δpanc^ mice (Huang, Snider, & Cross, 2008; Yamashita et al., 2001; Zhang et al., 2010). Current genetic mouse models of GDM exhibit characteristics of the human disease including impaired glucose tolerance and a decreased β‐cell proliferation. However, the db/+ mouse, for example, shows hyperphagia and severe obesity, which is not evident in humans. Single‐gene mutations are suitable for the identification of pathways underlying GDM, but often represent simplified models, as they do not adequately describe the complex interaction of the polygenic disease. Surgical, that is, partial or total pancreatectomy, and chemically induced diabetes due to the administration of streptozotocin or alloxan do not appropriately reflect the human disease because they lead to a permanent, nonphysiological, and irreversible state of diabetes with a severe increase in blood glucose (Pasek & Gannon, 2013). Therefore, an unmet need exists for models which display the full range and the transient nature of the metabolic abnormalities.

To close this gap, our approach was to investigate the suitability of a polygenic mouse model, the New Zealand obese (NZO) mouse, as a potential model for human GDM. Early studies of male NZO revealed hyperglycemia, impaired glucose tolerance, hyperinsulinemia, and reduced insulin response following glucose stimulation (Larkins, 1973; Larkins, Simeonova, & Veroni, 1980). Numerous studies using male NZO mice have resulted in a well‐established model of polygenic obesity and T2DM (Kluge, Scherneck, Schürmann, & Joost, 2012). However, female mice of this strain are protected from hyperglycemia and β‐cell death due to the sex hormone estrogen. But glycemic control of female NZO impairs following long‐term administration of high‐fat diet or after ovarectomy and animals develop a disease pattern of T2DM similar to that of humans (Lubura et al., 2015; Vogel et al., 2013).

There is strong evidence that GDM and T2DM share a common pathophysiology, same risk factors like adiposity and a family history of diabetes, and a common genetic background (Cho et al., 2009; Huopio et al., 2013; Robitaille & Grant, 2008). Therefore, it is plausible that female NZO mice may be considered as a suitable polygenic model to study the pathophysiology of GDM, which was the aim of this study. In particular, it should be shown whether the NZO mouse exhibits impaired glucose tolerance before and/or during pregnancy and to what extent insulin secretion is impaired. Moreover, we wanted to investigate whether the phenotype of female NZO mice can be assigned to one of the different subtypes of human GDM or to a preconceptional metabolic disorder influenced by pregnancy.

## 2 MATERIAL AND METHODS

### 2.1 Animals

All experiments were approved by the ethics committee of the Lower Saxony State Office for Consumer Protection and Food Safety (Oldenburg, Germany; ethics approval number: 33.19‐42502‐04‐17/2462; internal ID (05.15) TSB TU BS). Breeding pairs of the NZO (NZO/HIBomDife) and the NMRI (NMRI/RJ) control strain were provided from the German Institute of Human Nutrition (Nuthetal, Germany) and further kept and mated in an own breeding. This strain was chosen as a suitable control, since female NMRI mice represent a control with high body weight without diabetes and have a large litter size, which results in a high stimulation of Langerhans islets during pregnancy. Moreover, it represents a robust model of normal β‐cell physiology. Mice were housed in an air‐conditioned room at 21 ± 1°C with a 12:12 hr light‐dark cycle (lights on at 06:30 am). Animals had ad libitum access to water and food (1328 P, Altromin) with a content of 11% fat, 24% protein, and 65% carbohydrates with a total metabolizable energy of 13.5 kJ/g. Female NZO and NMRI mice were mated over night at the age of about 7 weeks. Pregnancy was confirmed by examining vaginal plugs the following morning. This day was considered as day 0.5 and mice were studied on day 14.5 of gestation at the age of about 9 weeks. The preconceptionally examined group was of the same age as the pregnant one. These animals were used for perifusion experiments, measurement of plasma and pancreatic hormone concentrations, and histological analyses (see below). For in vivo experiments, the same animals throughout were studied over the period of time specified below to determine alterations of blood glucose levels, glucose tolerance, insulin secretion, and sensitivity. Postpartum OGTT was performed about 4 weeks after birth of offspring. Lactation lasted 3 weeks and the offspring were weaned after this period. Different animals were used for the individual experiments, that is, OGTT, immunohistochemistry, total pancreatic hormone content, and islet isolation.

### 2.2 Oral glucose tolerance tests (OGTT) and determination of in vivo insulin secretion

After 6 hr of food deprivation, basal blood glucose, plasma insulin concentrations, and body weights of NZO and NMRI mice were determined. Each animal received an oral glucose bolus of 2 mg glucose/ g body weight by gavage. These OGTT conditions were recommended to achieve robust differences in blood glucose and plasma insulin concentrations (Andrikopoulos, Blair, Deluca, Fam, & Proietto, 2008). At time points 7.5, 15, 30, 60, and 120 min after application, blood glucose was measured from the tail tip using a glucose meter (Contour next, Ascensia Diabetes Care). At each time point, blood samples were collected from tail tip into heparin‐coated microtubes (Sarstedt) and kept on ice. After centrifugation, plasma samples were stored at −80°C for later insulin measurement. Keeping stress as low as possible, mice were kept in their usual cages during the test and had free access to water.

### 2.3 Plasma hormone concentrations

Random plasma hormone concentrations were measured in animals fed ad libitum and postabsorptive concentrations after 6 hr of food deprivation. Blood was collected via the vena cava or the heart in deep anesthesia followed by a cervical dislocation. After centrifugation, plasma samples were stored at −80°C for later measurements. Plasma insulin concentrations were determined by Mouse Ultrasensitive Insulin ELISA according to manufacturer's protocol (ALPCO). Plasma glucagon concentrations were determined by Glucagon ELISA (Mercodia).

### 2.4 Indices of insulin sensitivity

Homeostasis model assessment of insulin resistance (HOMA‐IR), a surrogate marker of insulin resistance, was calculated as (fasting glucose concentration x fasting insulin concentration)/ 22.5 (Matthews et al., 1985). To evaluate whole body insulin sensitivity from data obtained during oral glucose tolerance test, Matsuda index was calculated as (10,000/ √ (fasting insulin × fasting glucose × mean insulin during OGTT × mean glucose during OGTT)) (Matsuda & DeFronzo, 1999).

### 2.5 Total pancreatic hormone content

Tissues were collected from animals fed ad libitum after cervical dislocation. After sampling, pancreas was weighed, quickly frozen in liquid nitrogen, and stored at −80°C for further processing. Whole pancreas was homogenized in ice‐cold acid ethanol (0.18 M HCl in 70% ethanol) and incubated over night at 4°C. After centrifugation (5,000 g, 15 min, 4°C), supernatants were stored at −20°C for later measurements using Mouse High Range Insulin ELISA (ALPCO) and Glucagon ELISA (Mercodia) according to manufacturer's protocols.

### 2.6 Immunohistochemistry and immunofluorescence of pancreatic sections

Pancreatic tissues were obtained from animals fed ad libitum after cervical dislocation and fixed in 4% phosphate buffered formaldehyde for 24 hr. Fixed tissues were then embedded in paraffin according to standard procedures. Serial sections of 4 μm at sampling intervals of 150 μm were prepared. After rehydration, immunohistochemical staining was carried out on three sections, using rabbit polyclonal anti‐glucagon antibody (1:200; Cell Marque), rabbit polyclonal anti‐somatostatin antibody (1:2,000; Abcam), and rabbit monoclonal anti‐Ki‐67 antibody (1:250; Cell Marque). Prior to staining of Ki‐67 antibody, heat‐mediated antigen retrieval was performed, using a citrate buffer at pH 6.0 (Dako REAL^TM^ Target Retrieval Solution; Dako, Agilent Technologies). Secondary antibody was Histofine Simple Stain Mouse MAX PO anti‐rabbit (Nichirei Biosciences), detected by diaminobenzidine (Dako) according to manufacturer's instructions. Nuclei were then stained with Mayer's hematoxylin. The microscopic evaluation was carried out on basis of three sectional planes per animal. To determine glucagon and somatostatin areas, one hundred islets per strain and condition (*n* = 4–6 animals per group) were randomly selected. Evaluation of glucagon and somatostatin area was performed with the upright microscope Eclipse Ni‐E (Nikon), equipped with the DS‐Fi3 Color Camera (Nikon) and analysis software NIS elements AR 5 (Nikon). Evaluation of Ki‐67‐positive islet cells was performed with the upright microscope Leitz Orthoplan (Leitz), equipped with VisiCAM 100 (Visitron Systems GmbH) and the software VisiVIEW (Visitron Systems GmbH). When evaluating Ki‐67, all islets of three sectional planes per animal (*n* = 5–6 animals per group) were considered and manual counting of Ki‐67‐positive nuclei was conducted using ImageJ software (Rasband, 2011). In addition, double immunofluorescence staining for insulin (mouse monoclonal anti‐insulin antibody; 1:50,000; Sigma‐Aldrich) and glucagon (1:100, Cell Marque) was performed on representative pancreatic slices. Primary antibodies were detected with fluorophore‐labeled secondary antibodies using Rhodamine Red‐X goat anti‐mouse (1:200, Jackson ImmunoResearch) and Alexa Fluor488 goat anti‐rabbit (1:500, Jackson ImmunoResearch) plus DAPI (KPL). Acquisition of representative images was performed with the upright microscope Eclipse Ni‐E (Nikon), equipped with the DS‐Fi3 Color Camera (Nikon) and analysis software NIS elements AR 5 (Nikon).

### 2.7 Glucose‐stimulated insulin secretion of primary mouse islets

Islets were isolated by a collagenase digestion technique from animals fed ad libitum after cervical dislocation (Collagenase P, Roche; 0.5 mg/ml) in HEPES‐buffered Krebs‐Ringer medium (115 mM NaCl, 4.7 mM KCl, 2.6 mM CaCl_2_, 1.2 mM KH_2_PO_4_, 1.2 mM MgSO_4_, 20 mM NaHCO_3_, 10 mM HEPES, and 2 mg/ml BSA) which was saturated with 95% O_2_ and 5% CO_2,_ containing 5 mM glucose. Briefly, the pancreas was inflated by injection of 3 ml collagenase solution into the common bile duct. Subsequently, the pancreas was digested for 8:45 min in a water bath at 37°C and shaken vigorously for 1 min. Digestion was stopped by adding ice‐cold Krebs‐Ringer medium and washed twice with centrifugation steps in between (300 g, 15 s). Islets were passaged several times in Krebs‐Ringer medium to remove exocrine pancreatic parts by hand‐picking under the stereomicroscope. Fifty freshly isolated islets were then given into a purpose‐made perifusion chamber and perifused with a rate of 1 ml/min at a temperature of 37°C. The perifusion medium consisted of HEPES‐buffered Krebs‐Ringer medium with different glucose concentrations (5 and 20 mM). The efflux was collected at defined time intervals and insulin secretion was determined by Rat Insulin ELISA (Mercodia) (Hatlapatka, Willenborg, & Rustenbeck, 2009).

### 2.8 Statistical analysis

**FIGURE 1:**
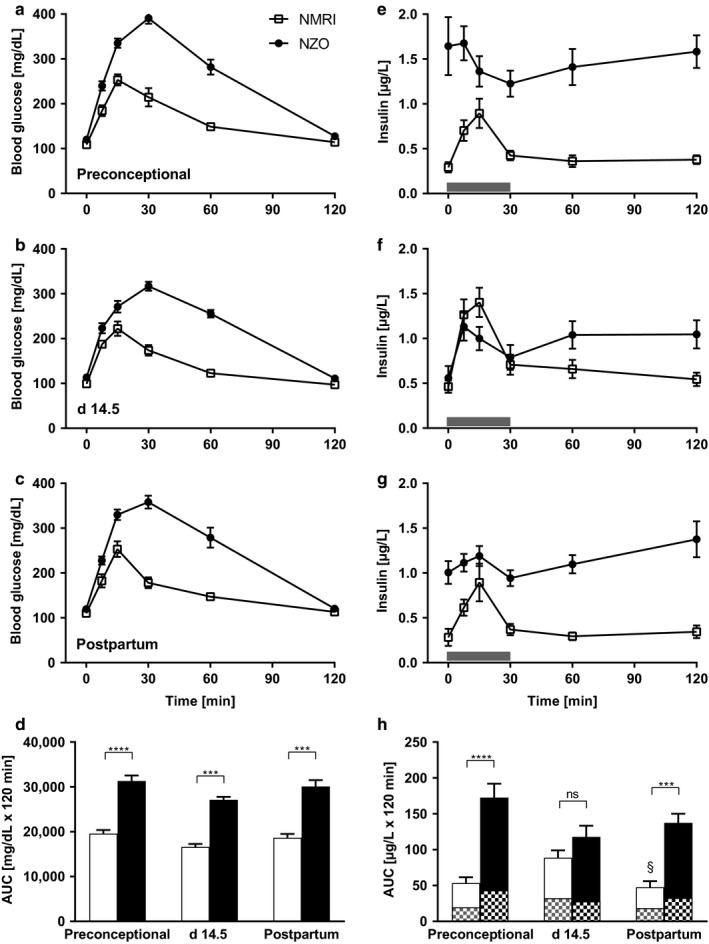
Impaired glucose tolerance and preconceptional hyperinsulinemia in female NZO mice. Blood glucose and plasma insulin concentrations during oral glucose tolerance tests of NZO mice (black circles) and NMRI control mice (white squares). (a, e) Preconceptional, (b, f) day 14.5 of gestation, and (c, g) postpartum. (d) Area under the curve (AUC) for blood glucose calculated using the trapezoidal rule of NZO mice (black bars) and NMRI control mice (white bars). (h) AUC for insulin secreted during OGTT of NZO and NMRI control mice. Squared parts of the bars indicate first phase (0–30 min) of insulin secretion. Data are presented as means ± *SEM* (*n* = 7–11 animals per group). ***p < .001, ****p < .0001, NMRI postpartum vs. day 14.5: §*p* = .0183

**FIGURE 2:**
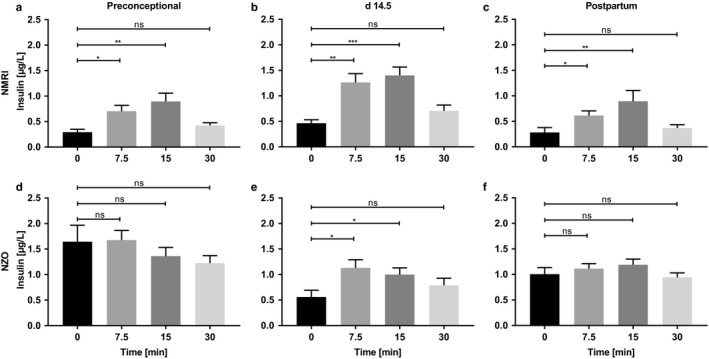
Detailed bar chart of Figure 1e–g highlighting the improved stimulation of insulin secretion in NZO mice during pregnancy. Insulin secretion during the first 30 min of OGTT at time points (a, d) preconceptional, (b, e) day 14.5 of gestation, and (c, f) postpartum of NMRI (upper graphs) and NZO mice (lower graphs). Data are presented as means ± *SEM* (*n* = 7–11 animals per group). *p < .05, **p < .01, ***p < .001

**FIGURE 3:**
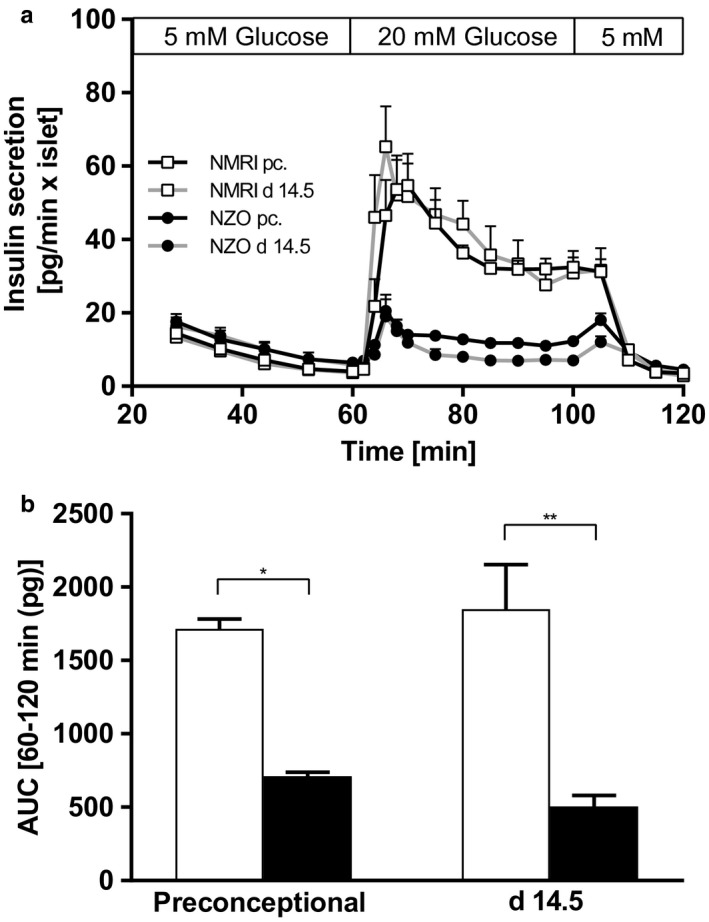
Insulin secretion defect of freshly isolated NZO islets. (a) Insulin secretion at time points preconceptional and day 14.5 of gestation of NZO mice (preconceptional: black line, black circles; d 14.5: grey line, black circles) and NMRI control mice (preconceptional: black line, white squares; d 14.5: grey line, white squares). Islets were perifused from 0 min to 60 min with medium containing 5 mM glucose. Then glucose concentration was raised to 20 mM for 40 min of perifusion, followed by a wash out period of 20 min at 5 mM glucose. (b) Area under the curve (AUC) was calculated using the trapezoidal rule (NZO: black bars; NMRI: white bars). Data are presented as means ± *SEM* (*n* = 4–5 animals per group). *p < .05, **p < .01

**FIGURE 4:**
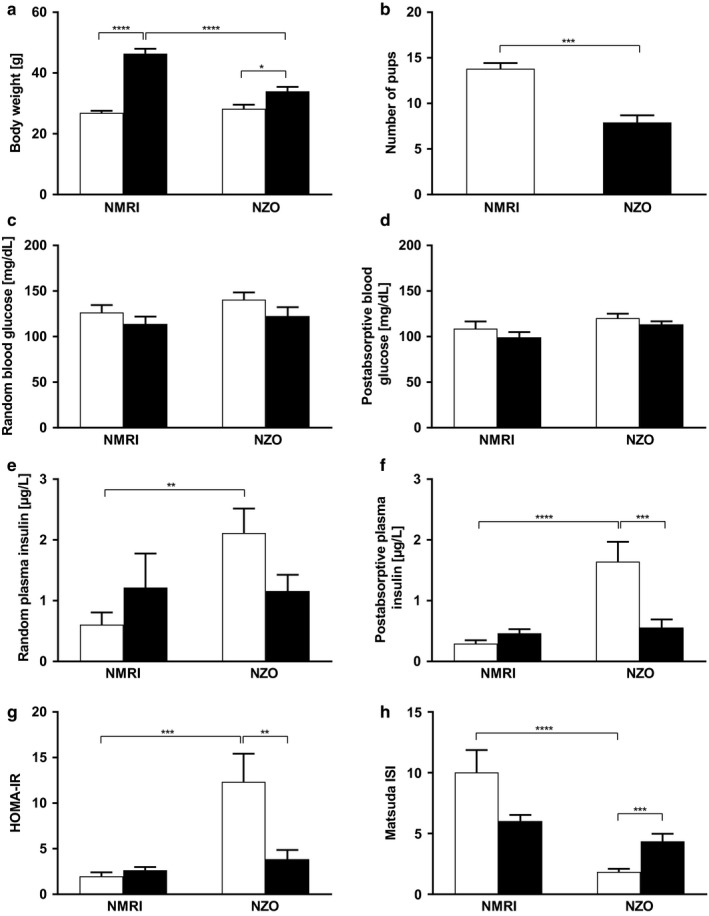
Insulin resistance without manifest diabetes in female NZO mice. (a) Body weights of NZO and NMRI control mice at time points preconceptional (white bars) and day 14.5 of gestation (black bars). (b) Number of pups of NZO (black bars) and NMRI control mice (white bars). (c) Random and (d) postabsorptive (6 hr food deprivation) blood glucose of NZO and NMRI control mice at time points preconceptional (white bars) and day 14.5 of gestation (black bars). (e) Random and (f) postabsorptive plasma insulin concentrations of both strains. (g) Calculation of insulin resistance by the homeostasis model assessment method (HOMA‐IR) and (h) Matsuda index of insulin sensitivity (ISI) of NZO and NMRI control mice. Data are presented as means ± *SEM* (a and b *n* = 10, c and e *n* = 5–7, d, f, g, and h *n* = 7–11 animals per group). *p < .05, **p < .01, ***p < .001, ****p < .0001

**FIGURE 5:**
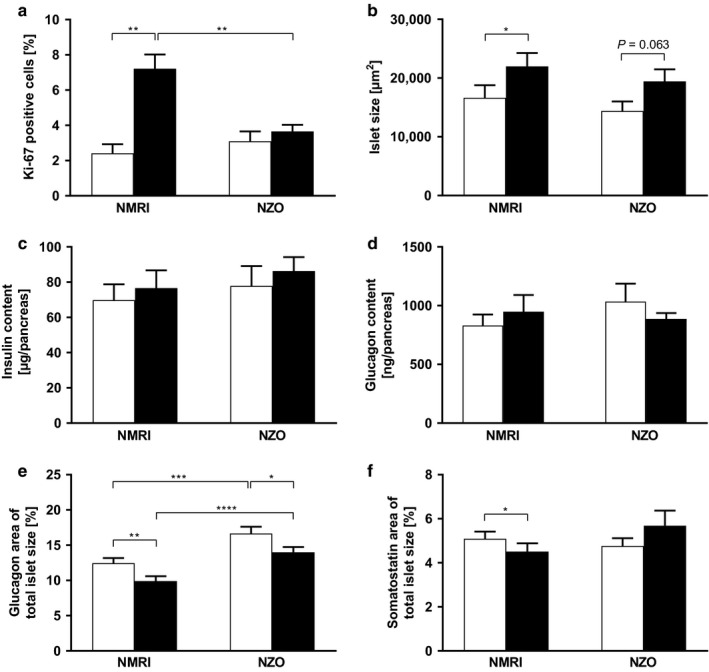
Different adaptation of Langerhans islets in NZO and NMRI mice during gestation. (a) Mean percentage of islet cells positive for Ki‐67 and (b) islet size of NZO and NMRI control mice at both time points, preconceptional (white bars), and on day 14.5 of gestation (black bars). (c) Total pancreatic insulin and (d) glucagon contents were determined in acid‐ethanol extracts. (e) Mean percentage of islet cells positive for glucagon and (f) somatostatin. Data are presented as means ± *SEM* (*n* = 4–7 animals per group). *p < .05, **p < .01, ***p < .001, ****p < .0001

For statistical analysis and graphical presentation GraphPad Prism 7 (GraphPad) was used. Data are presented as means ± *SEM*. Nonparametric Mann–Whitney *U* test was applied to compare means of both mouse strains separately for each of the time points or to compare differences within one strain over the period of time (e.g., NMRI preconceptional vs. d 14.5). This test was used for the following parameters: Figure 3b: AUC of insulin secreted during perifusion. Figure 4a: body weight, b: number of pups, c: random blood glucose, d: postabsorptive blood glucose, e: random plasma insulin, f: postabsorptive plasma insulin, g: HOMA‐IR, and h: Matsuda ISI. Figure 5a: Ki‐67‐positive cells, b: islet size, c: insulin content, d: glucagon content, e: glucagon area, and f: somatostatin area. Figure 7a: random plasma glucagon and b: postabsorptive plasma glucagon. To evaluate alterations within one strain over the period of time (e.g., NMRI preconceptional vs. d 14.5 vs. postpartum) and alterations of insulin secretion during OGTT, Kruskal–Wallis *H* test followed by Dunn's multiple‐comparison test was used for the following parameters: Figure 1d: AUC of blood glucose and h: AUC of insulin secreted during OGTT. Figure 2a‐f: insulin secretion during the first 30 min of OGTT. A value of *p* < .05 was considered statistically significant. P values were indicated as *p < .05, **p < .01, ***p < .001, and ****p < .0001.

## 3 RESULTS

### 3.1 Impaired glucose tolerance but improved responsiveness of islets of Langerhans in pregnant NZO mice

To determine glucose tolerance in vivo (Figure 1a‐c), mice were deprived of food for 6 hr and an OGTT was performed administering 2 mg glucose/g body weight. NZO mice showed impaired glucose tolerance with increased blood glucose excursions after stimulus compared with the control strain preconceptionally, on day 14.5 of gestation and postpartum. This is also reflected in significant increases in the AUC (Figure 1d) (NZO vs. NMRI; preconceptional: 31,321 ± 1,218 vs. 19,594 ± 784 mg/dl × 120 min, *p *= <.0001; day 14.5: 27,106 ± 662 vs. 16,627 ± 651 mg/dl × 120 min, *p* = .0002; postpartum: 30,087 ± 1,413 vs. 18,658 ± 854 mg/dl × 20 min, *p* = .0005). In addition, plasma samples were taken to determine insulin secretion in vivo. Insulin secretion was not stimulated in NZO mice by glucose preconceptionally (Figure 1e and Figure 2d), while the same glucose challenge had a significantly insulinotropic effect during pregnancy (Figure 1f and Figure 2e). This was associated with significant decreases in the fasting insulin values (NZO preconceptional vs. day 14.5: 1.65 ± 0.32 vs. 0.56 ± 0.13 µg/L, *p* = .0008). Postpartum, basal insulin secretion increased again in NZO mice and was not stimulated by glucose (Figure 1g and Figure 2f). Compared with the NZO strain, NMRI control mice showed a significant increase in insulin secretion after glucose application at all three time points. During pregnancy this was even more pronounced (Figure 2a‐c). Total amount of insulin secreted during OGTT, expressed as AUC, was increased in the NZO strain at all three time points (Figure 1h) (NZO vs. NMRI; preconceptional: 172.7 ± 19.3 vs. 53.5 ± 8.0 µg/l × 120 min, *p *= <.0001; day 14.5: 117.7 ± 15.7 vs. 88.8 ± 10.3 µg/l × 120 min, ns; postpartum: 137.4 ± 12.7 vs. 47.7 ± 8.5 µg/l × 120 min, *p* = .0006).

### 3.2 Isolated Langerhans islets of NZO mice did not respond adequately to a glucose stimulus

To investigate insulin secretion under exclusion of systemic influences, primary Langerhans islets were isolated and kinetics were determined in perifusion experiments (Figure 3a). Compared with NZO, NMRI islets secreted significantly higher amounts of insulin at both time points. During pregnancy, increase in insulin secretion after stimulus was prompt and showed a steeper increase within this strain. Preconceptionally, freshly isolated NZO mouse islets showed weak secretory response to the increase in glucose from 5 mM to 20 mM. In contrast to the in vivo experiments, this reduced responsiveness was unchanged on day 14.5 of gestation. Prestimulatory values of insulin secretion after 60 min of perifusion with medium containing 5 mM glucose were not significantly different at both time points (NZO vs. NMRI, preconceptional: 6.45 ± 1.40 vs. 4.06 ± 0.34 pg/min × islet, ns; day 14.5 of gestation: 5.86 ± 0.70 vs. 3.71 ± 0.38 pg/min × islet, ns). Total amount of insulin released during stimulation for 60 min, presented as AUC (Figure 3b), was significantly higher within the NMRI strain (NZO vs. NMRI, preconceptional: 711 ± 28 vs. 1,715 ± 68 pg × 60 min, *p* = .029; day 14.5 of gestation: 506 ± 75 vs. 1,849 ± 305 pg × 60 min, *p* = .008). During pregnancy, a slight but not significant decrease in insulin secretion was observed compared with preconceptional findings within the NZO strain (*p* = .06). This was primarily due to a significantly reduced second phase of insulin secretion, whereas this phase remained almost unchanged within the control strain.

### 3.3 Pregnancy improved preconceptional hyperinsulinemia of NZO mice

Preconceptionally, body weights were comparable between NZO mice and the NMRI control strain. During pregnancy, a substantial weight gain was observed in the control strain, whereas body weights of NZO mice increased only moderately (Figure 4a). This was due to a higher number of offspring in NMRI control mice. The average litter size in NMRI was 14 and 8 in NZO mice (Figure 4b). Birth weights of the offspring of both strains were comparable (data not shown). Random blood glucose levels were determined at time points preconceptional and on day 14.5 of gestation. The NZO mouse showed no overt hyperglycemia at any time point (Figure 4c). During pregnancy, blood glucose concentrations were slightly but not significantly lower than preconceptionally in both strains. Postabsorptive blood glucose concentrations (Figure 4d) were also not significantly different between the strains and slightly lower during pregnancy. Preconceptionally, random plasma insulin concentrations (Figure 4e) were significantly higher in NZO mice compared with the control (NZO vs. NMRI: 2.1 ± 0.4 vs. 0.6 ± 0.2 µg/L, *p* = .004). During pregnancy, random plasma insulin concentrations slightly increased in control mice and decreased in NZO mice (NZO vs. NMRI: 1.2 ± 0.3 vs. 1.2 ± 0.6 µg/L, ns). Postabsorptive insulin concentrations (Figure 4f) were similar in their profiles. Preconceptional hyperinsulinemia of the NZO was more pronounced, improving significantly during pregnancy (NZO preconceptional vs. day 14.5: 1.6 ± 0.3 vs. 0.6 ± 0.1 µg/L, *p* = .0008). During OGTT, insulin secretion of NZO increased notably after the first phase, indicating insulin resistance. This observation was supported by calculating the HOMA‐IR (Figure 4g) and Matsuda index for insulin sensitivity (Figure 4h). Preconceptionally, HOMA and Matsuda indices differed significantly between the two strains, implying increased insulin resistance (NZO vs. NMRI: 12.34 ± 3.09 vs. 1.98 ± 0.44, *p* = .0001) and decreased insulin sensitivity of the NZO (NZO vs. NMRI: 1.85 ± 0.25 vs. 10.03 ± 1.84, *p *= <.0001). During pregnancy, HOMA and Matsuda indices did not differ between NZO and NMRI mice (NZO vs. NMRI: HOMA: 3.86 ± 0.99 vs. 2.63 ± 0.37, ns; Matsuda: 4.36 ± 0.63 vs. 6.03 ± 0.50, ns), since insulin resistance decreased and insulin sensitivity increased significantly in NZO. Within the NMRI control strain insulin resistance increased and insulin sensitivity decreased slightly but not significantly, reflecting the natural adaptation during pregnancy.

### 3.4 Improved responsiveness during pregnancy was not a cause of enhanced islet cell proliferation

**FIGURE 6:**
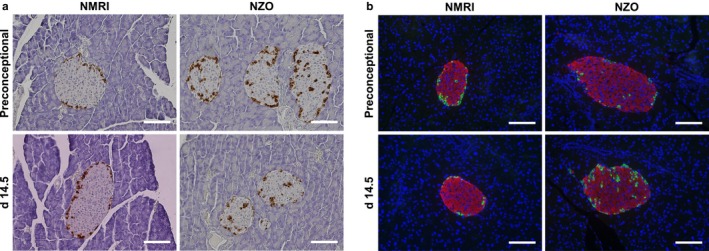
Different α‐cell distribution in female NZO mice. (a) Representative images showing glucagon‐positive islet cells (brown) of NZO and NMRI control mice at time points preconceptional and on day 14.5 of gestation. (b) Representative images of a double immunofluorescence staining for insulin (red) and glucagon (green) at time points preconceptional and on day 14.5 of gestation. Nuclei were stained with DAPI (blue). Scale bars: 100 µm

To determine adaptive changes in Langerhans islets during pregnancy, pancreata were examined immunohistologically at time points preconceptional and on day 14.5 of gestation. The proliferation marker Ki‐67 (Figure 5a) was used to determine the proliferative response of Langerhans islets to the increased insulin demand during pregnancy. Preconceptionally, Ki‐67‐positive islet cells were not significantly different between the strains (NZO vs. NMRI: 3.1 ± 0.6 vs. 2.4 ± 0.5%, ns). On day 14.5 of gestation, islet cells of NZO mice showed significantly lower proliferation in comparison to the NMRI control strain (NZO vs. NMRI: 3.7 ± 0.4 vs. 7.2 ± 0.8%, *p* = .004). So, in contrast to NZO, a significant increase in proliferation could be observed during pregnancy in the NMRI control strain (2.4 ± 0.5 vs. 7.2 ± 0.8%, *p* = .002). Mean islet size (Figure 5b) of NZO and NMRI control mice was not significantly different at both time points. During pregnancy, islet size increased within the NMRI control strain (16,601 ± 2,162 vs. 21,987 ± 2,276 µm^2^, *p* = .031). Also in NZO mice islet size increased slightly, but not significantly (14,382 ± 1,635 vs. 19,437 ± 2,062 µm^2^, *p* = .063). To estimate β‐cell mass, total pancreatic insulin content (Figure 5c) was determined in acid‐ethanol extracts. At both time points, insulin contents did not differ significantly between the two strains, but were moderately higher in NZO mice. During pregnancy, insulin contents increased slightly in both strains. Preconceptionally, total pancreatic glucagon content (Figure 5d) was slightly higher in NZO mice, decreased insignificantly on day 14.5 of gestation within this strain, but increased slightly in NMRI control mice (NZO: 1,034 ± 153 vs. 887 ± 50 ng/pancreas, ns; NMRI: 831 ± 93 vs. 948 ± 143 ng/pancreas, ns). The percentage of glucagon positive area of total islet size (Figure 5e) was markedly increased within the NZO at both time points. In NZO and NMRI control mice, glucagon‐positive area decreased significantly during pregnancy (NZO: 16.7 ± 0.9 vs. 14.0 ± 0.8%, *p* = .041; NMRI: 12.5 ± 0.7 vs. 9.9 ± 0.7%, *p* = .001). Comparing the somatostatin‐positive area (Figure 5f), no significant differences between both strains were observed. In NZO mice, somatostatin increased slightly but not significantly during pregnancy (4.8 ± 0.4 vs. 5.7 ± 0.7%, *p* = .95) and decreased significantly in NMRI mice (5.1 ± 0.3 vs. 4.5 ± 0.4%, *p* = .018). Immunohistochemistry and immunofluorescence revealed a differing α‐cell distribution pattern in Langerhans islets between both strains. In NZO mice, α‐cells were located peripherally as well as randomly in the core of the islet. The NMRI control strain showed a peripheral distribution pattern of α‐cells (Figure 6a,b).

### 3.5 Pregnancy improved preconceptional hyperglucagonemia in NZO mice

**FIGURE 7:**
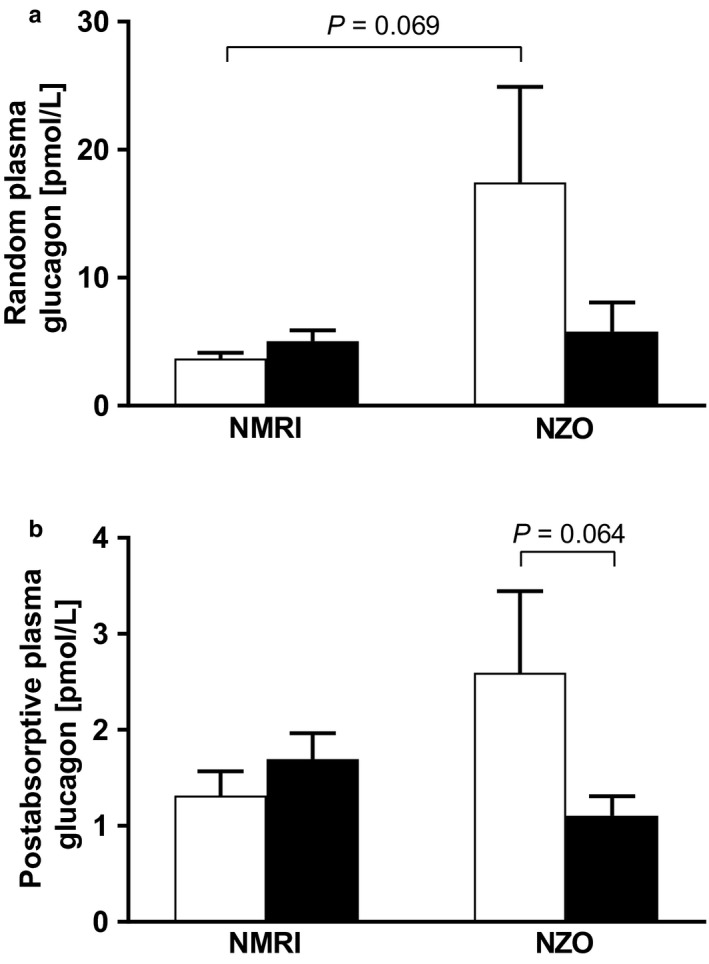
Preconceptional hyperglucagonemia in female NZO mice. (a) Random and (b) postabsorptive (6 hr food deprivation) plasma glucagon concentrations of NZO and NMRI control mice at both time points, preconceptional (white bars) and on day 14.5 of gestation (black bars). Data are presented as means ± *SEM* (*n* = 5–7 animals per group)

Random plasma glucagon levels (Figure 7a) revealed preconceptional hyperglucagonemia of the NZO mouse (NZO vs. NMRI: 17.44 ± 7.47 vs. 3.68 ± 0.46 pmol/L, *p* = .069). Postabsorptive glucagon concentrations (Figure 7b) were similar in their profiles. A slight increase within the control strain during pregnancy was observed, whereas levels decreased slightly but not significantly in NZO mice (preconceptional vs. day 14.5, NZO: 2.60 ± 0.85 vs. 1.11 ± 0.20 pmol/l, *p* = .064; NMRI: 1.31 ± 0.25 vs. 1.69 ± 0.27 pmol/l, ns).

## 4 DISCUSSION

Adaptive mechanisms of Langerhans islets to an increased insulin demand during pregnancy, glucose tolerance, and insulin secretion profiles in vivo and ex vivo were evaluated in the polygenic NZO mouse model and compared with the NMRI control strain. The data describe the phenotype of female NZO mice prior to conception, during gestation, and postpartum and affirm that the NZO strain displays important characteristics of subpopulations of human GDM and/or prediabetes in pregnancy. This underlines the heterogeneity of the metabolic disease.

NZO mice showed no overt hyperglycemia but exhibited impaired glucose tolerance. Preconceptionally impaired glucose tolerance did not deteriorate during pregnancy and persisted postpartum. GDM, in the strict sense, is defined as impaired glucose tolerance with first recognition during pregnancy, diagnosed by 75‐g OGTT. Since in humans an OGTT is routinely performed only between the second and third trimester of pregnancy, data from preconceptional tests are rare and were often obtained from cohorts with specific risk factors (Catalano et al., 1993). Limited data are available from women affected by polycystic ovarian syndrome (PCOS) or fertility disorders and the desire to become pregnant (Lanzone et al., 1996; Wei et al., 2008, 2017). It is known that women having PCOS represent a heterogeneous subgroup which is at higher risk to develop impaired glucose tolerance, both preconceptionally and during pregnancy (Lanzone et al., 1996). There is an association between preconceptional impaired glucose tolerance and adverse pregnancy outcome, independent from BMI (Wei et al., 2017). Furthermore, it could be shown that preconceptionally elevated plasma insulin concentrations increase the risk of glycemic disorders during pregnancy, although PCOS patients significantly increased secretion to compensate for higher insulin requirement (Lanzone et al., 1995). When screening for women with early manifestation of GDM, it was found that these women had elevated AUCs of glucose and insulin during OGTT compared with women with late manifestation or normal glucose tolerance (Bozkurt et al., 2015). Also NZO mice showed early hyperinsulinemia, impaired glucose tolerance, and insulin secretion could not be stimulated by glucose. During pregnancy, fasting insulin values were significantly lower and comparable with the values of the NMRI control strain. In addition, the application of glucose had a significantly insulinotropic effect in female NZO with a distinct first phase of insulin secretion, which could not be observed prior to conception. AUC of first phase insulin secretion during OGTT between both strains was comparable during pregnancy. Besides significantly reduced hyperinsulinemia, an improvement of insulin resistance and sensitivity during pregnancy of the NZO strain, was found.

In contrast to in vivo experiments, freshly isolated primary islets of the NZO strain showed weak secretory response in perifusion experiments, both preconceptionally and on day 14.5 of gestation. AUC of insulin secreted during perifusion experiments was significantly higher in NMRI control mice at both time points. Compared with the in vivo experiments, an improvement of insulin secretion could not be observed ex vivo. It is reasonable to assume that extrapancreatic modulators such as pregnancy hormones or the interaction of peripheral organs have a beneficial effect on insulin secretion and glucose homeostasis. Besides pregnancy hormones, changes in cytokine concentrations also affect insulin sensitivity. Alterations in pro‐ and anti‐inflammatory cytokine concentrations, such as adiponectin, leptin, and TNFα, have been observed in women affected by GDM (Fasshauer, Blüher, & Stumvoll, 2014; Wedekind & Belkacemi, 2016). In humans, increased TNFα and decreased adiponectin serum concentrations are associated with GDM (Cseh et al., 2004; Guillemette et al., 2014; Kinalski, Telejko, Kuzmicki, Kretowski, & Kinalska, 2005). Since adipose tissue, which secrets TNFα, is elevated in NZO mice, influences of cytokines on insulin secretion would also be conceivable and provide an approach for further investigations.

The most important finding of histological analysis was a significant increase in islet cell proliferation solely in the NMRI control strain, whereas proliferation remained almost unchanged in NZO islets. Despite the proliferation defect, in vivo insulin response improved during pregnancy in NZO mice. Which role pregnancy‐associated hormones may have is arguable, as increased insulin secretion and proliferation correlate with their secretion (Parsons, Brelje, & Sorenson, 1992; Sorenson & Brelje, 1997). NZO mice showed no differences in plasma prolactin concentrations compared with the control strain both preconceptionally and during pregnancy (data not shown). For this reason, improved responsiveness of the NZO mouse cannot be solely explained by this pregnancy‐associated hormone and the underlying mechanisms have to be further investigated. Changes in insulin content cannot be a reason either, as this remains unaffected by pregnancy, with a trend toward elevated levels in the NZO strain at both time points.

In male NZO mice, a model of T2DM, it was shown that under glucotoxic conditions proliferation of β‐cells could not be initiated in comparison to diabetes‐resistant B6‐ob/ob and candidate genes of this defect were identified (Kluth et al., 2015). Even in healthy women, pregnancy is a diabetogenic condition associated with reduced insulin sensitivity and therefore requires a compensatory increase in β‐cell mass and function (Di Cianni et al., 2003). Since T2DM and gestational diabetes have similar genetic reasons (Cho et al., 2009; Huopio et al., 2013; Robitaille & Grant, 2008), it is conceivable that this proliferation defect of male NZO is also present in female animals.

The physiologically increasing insulin resistance during pregnancy facilitates the supply of energy to the fetus and therefore insulin requirements rise continuously (Lain & Catalano, 2007). Functionally effective β‐cell mass increases as a result of hyperplasia and hypertrophy. These adaptive mechanisms during pregnancy are regulated by lactogenic hormones such as placental lactogen and prolactin (Parsons et al., 1992; Sorenson & Brelje, 1997). In addition, serotonin, which is produced and secreted in high concentrations during pregnancy, regulates β‐cell proliferation and inhibition of its synthesis leads to impaired glucose tolerance (Kim et al., 2010). An increase in islet area during pregnancy could also be shown in the strains examined here, albeit to a greater extent in the NMRI control strain. This was associated with a significant decrease in the percentage of glucagon and somatostatin areas in the NMRI control strain. Due to the expansion of islet area, with simultaneous reduction in α‐ and δ‐cell proportions, it can be assumed that functional β‐cell area increased as an adaptive mechanism. There was only a significant decrease in glucagon area within the NZO strain, whereas somatostatin area increased slightly but not significantly. These observations imply an increase in β‐cell area, although of lower magnitude than in the control strain. There was an interesting observation of the α‐cell distribution pattern in Langerhans islets within the NZO strain. α‐cells were located peripherally as well as randomly in the core of the islet, whereas the control strain showed a peripheral distribution pattern. A peripheral α‐cell distribution reflects the normal physiology of mouse islets. However, increased insulin demands, as known from prediabetes or insulin resistance, lead to an occurrence of α‐cells inside the core. An improved vascularization with more efficient cell–cell communication and thus an improved sensitivity for changes in glucose concentration are possible underlying reasons (Kharouta et al., 2009; Steiner, Kim, Miller, & Hara, 2010). These early alterations in islet architecture could therefore influence the prediabetic metabolism with preconceptional hyperinsulinemia, hyperglucagonemia, and insulin resistance in NZO mice. It is known that glucagon stimulates insulin secretion by activation of glucagon and GLP‐1 receptors on β‐cells (Kawai, Yokota, Ohashi, Watanabe, & Yamashita, 1995; Svendsen et al., 2018). We observed a trend toward a decline in plasma and total pancreatic glucagon concentrations during pregnancy in NZO mice. This could also affect preconceptional hyperinsulinemia, which improved during pregnancy.

In summary, our data indicate that NZO mice exhibit important characteristics of human gestational diabetes. Nonetheless, due to the heterogeneity of the disease, only certain subpopulations may be represented. Patients with early alterations in glucose metabolism, impaired glucose tolerance, and lack of compensatory adaptation of Langerhans islet should be emphasized here. Furthermore, patients already suffering from prediabetes prior to pregnancy and diagnosed as GDM patients during pregnancy can be considered. Glucose‐stimulated insulin secretion differed significantly between in vivo experiments, in which responsiveness of insulin secretion improved during pregnancy, and ex vivo experiments without any change in secretion. The underlying mechanisms, in which pregnancy‐associated hormones such as serotonin and cytokines can be of decisive importance, are subject of further investigation.

## CONFLICT OF INTEREST

All authors declare no conflicts of interest relevant to this work.

## AUTHOR CONTRIBUTIONS

K.G. and S.S. conceived and designed research. K.G., M.A.P., F.D., and M.L. performed experiments. K.G. analyzed data and prepared figures. K.G., I.R., and S.S. interpreted results of experiments. K.G. and S.S. drafted manuscript. I.R. and A.S. revised the manuscript. K.G., M.A.P., F.D., M.L., I.R., A.S., and S.S. approved final version of manuscript.
